# Consensus genomic regions and key genes for biotic, abiotic and key nutritional traits identified using meta- QTL analysis in peanut

**DOI:** 10.3389/fpls.2025.1539641

**Published:** 2025-04-15

**Authors:** Aakash Sahu, Sagar Krushnaji Rangari, Yogesh Dashrath Naik, Anjali Jyotish, Manish K. Pandey, Rajeev K. Varshney, Mahendar Thudi, Somashekhar M. Punnuri

**Affiliations:** ^1^ Department of Agricultural Biotechnology and Molecular Biology, Dr. Rajendra Prasad Central Agricultural University (RPCAU), Pusa, Bihar, India; ^2^ Center of Excellence in Genomics & Systems Biology, International Crops Research Institute for the Semi-Arid Tropics (ICRISAT), Hyderabad, Telangana, India; ^3^ Western Australian (WA) State Agricultural Biotechnology Centre, Centre for Crop and Food Innovation, Murdoch University, Murdoch, WA, Australia; ^4^ College of Agriculture, Family Sciences and Technology, 1005 State University Dr, Fort Valley State University (FVSU), Fort Valley, GA, United States; ^5^ Centre for Crop Health, University of Southern Queensland (USQ), Toowoomba, QLD, Australia

**Keywords:** peanut, aflatoxin, biotic and abiotic stresses, meta-QTL, genes, yield

## Abstract

Peanut (*Arachis hypogaea* L.), a key oilseed crop in the U.S., plays a significant role in agriculture and the economy but faces challenges from biotic and abiotic stresses, including aflatoxin contamination caused by *Aspergillus flavus* and *A. parasiticus*. Despite many large-effect QTLs identified for yield and key traits, their use in breeding is limited by unfavorable genetic interactions. To overcome this, we aimed to identify consensus genomic regions and candidate genes linked to key traits by analyzing QTL data from 30 independent studies conducted over the past 12 years, focusing on biotic, abiotic, aflatoxin, morphological, nutritional, phenological, and yield-associated traits. Using genetic map information, we constructed consensus maps and performed a meta-analysis on 891 QTLs, leading to the identification of 70 Meta-QTLs (MQTLs) with confidence intervals ranging from 0.07 to 9.63 cM and an average of 2.33 cM. This reduction in confidence intervals enhances the precision of trait mapping, making the identified MQTLs more applicable for breeding purposes. Furthermore, we identified key genes associated with aflatoxin resistance in MQTL5.2 (serine/threonine-protein kinase, BOI-related E3 ubiquitin-protein ligase), MQTL5.3, MQTL7.3, and MQTL13.1. Similarly, for yield-related traits in MQTL3.1–MQTL3.4 (mitogen-activated protein kinase, auxin response factor), MQTL11.2 (MADS-box protein, squamosa promoter-binding protein), and MQTL14.1. Genes related to oil composition within MQTL5.2 (fatty-acid desaturase FAD2, linoleate 9S-lipoxygenase), MQTL9.3, MQTL19.1 (acyl-CoA-binding protein, fatty acyl-CoA reductase FAR1), MQTL19.4, and MQTL19.5. Nutritional traits like iron and zinc content are linked to MQTL1.1 (probable methyltransferase, ferredoxin C), MQTL10.1, and MQTL12.1. These regions and genes serve as precise targets for marker-assisted breeding to enhance peanut yield, resilience, and quality.

## 1 Introduction

Peanut (*Arachis hypogaea*), also known as groundnut or earthnut, is an allotetraploid oilseed legume crop (2n=4x=40) with a genome size of 2.7 Gb. It is the second most widely cultivated legume crop globally, primarily grown for oil production, following soybean (*Glycine max* L.; Vishwakarma et al., 2017). Peanuts are a key ingredient in ready-to-use therapeutic and supplementary foods aimed at combating malnutrition in underdeveloped and developing regions (Variath and Janila, 2017). Peanut seeds contain approximately 45–50% oil, 25% protein, 15% carbohydrates, and various beneficial secondary compounds. They also provide all 20 amino acids, making a significant contribution to human nutrition (Shasidhar et al., 2017; Wang et al., 2015).

Global peanut production reached 539 lakh tons, harvested from 327 lakh hectares, with an average productivity of 1,648 kg per hectare (FAOSTAT, 2021). China, India, Nigeria, the United States, and Sudan together account for about 69% of global peanut production (USDA, 2024; https://ipad.fas.usda.gov/cropexplorer/cropview/commodityView.aspx?cropid=2221000). In 2022–2023, India exported approximately 668,885.40 metric tons of peanuts (APEDA, 2024; https://apeda.gov.in/apedawebsite/GroundNut/GroundNut.htm). Notably, around 70% of the peanut cultivation area is located in arid and semi-arid regions. Peanuts are highly adaptable, thriving in soils with low chemical inputs, requiring minimal water, and exhibiting the lowest carbon footprint among nuts (Pandey et al., 2020). Additionally, peanut plants are zero-waste crops and play a role in preventing soil erosion (National Peanut Board, 2023; Agropedia, 2023).

Despite their significant economic and nutritional importance, peanut production faces substantial challenges from biotic and abiotic stresses, including pests, diseases, and adverse environmental conditions. One major constraint is aflatoxin contamination, which severely reduces peanut quality and poses serious health risks to humans (Pandey et al., 2017). Additionally, diseases such as tomato spotted wilt virus (TSWV), early leaf spot (ELS), late leaf spot (LLS), rust, root-knot nematode (RKN), and stem rot (SR) are critical biotic threats to peanut production worldwide. These challenges lead to significant yield losses and adversely affect the quality of peanut oil and seeds.

While both high and low oil content in peanuts have value—small seeds are preferred for oil production and large seeds for confectionery products (Wang et al., 2015; Gangurde et al., 2022)—a high oleic-to-linoleic acid ratio is particularly desirable. This trait improves shelf life and provides health benefits for both manufacturers and consumers (Wang et al., 2015; Pandey et al., 2014). Farmers and traders can benefit substantially from incorporating stable, high-oil content traits into elite cultivars, as even a 1% increase in oil content can raise producer profits by 7% (Liao, 2003). Many agronomic traits in peanuts are inherited quantitatively and influenced by genotype × environment interactions, highlighting the importance of identifying stable and promising genomic regions for crop improvement. Discovering such genomic regions, especially those conferring resistance to aflatoxins, is critical for sustainable peanut production (Yu et al., 2019; Variath and Janila, 2017).

The primary focus of peanut breeding is to improve yield, which is closely linked to pod, seed, and disease-related traits. However, these are complex quantitative traits that can be enhanced through quantitative trait loci (QTL) mapping (Lu et al., 2018). Over the two decades, numerous QTL studies on important traits have been conducted (PeanutBase, 2023; Ahmad et al., 2020; Dodia, 2018; Lu et al., 2018; Pandey et al., 2017; Bertioli et al., 2016; Sujay et al., 2012). However, many of these studies are limited to specific environments, leading to inconsistent and unstable phenotypes when these traits are introduced into elite cultivars with new genetic backgrounds. This instability often arises from unfavorable epistatic interactions (Lu et al., 2018). Consensus genomic regions and candidate genes play a pivotal role in the efficient transfer of desirable traits into crops, significantly reducing the risk of genetic load. Genetic load refers to the accumulation of unfavorable or non-beneficial alleles that can arise during traditional breeding approaches. By focusing on consensus genomic regions identified through meta-QTL (MQTL) analysis, breeders can target stable and well-defined loci associated with important traits, ensuring precision and efficiency in marker-assisted selection (MAS).

MQTL analysis has emerged as a powerful approach to refining and consolidating data from multiple QTL studies, thereby improving the precision and utility of genetic markers for breeding (Kumar et al., 2023; Isha et al., 2024; Gupta et al., 2024). While individual QTL studies provide valuable insights, they often report QTLs with large confidence intervals (CI) and inconsistencies across different environments and genetic backgrounds. MQTL analysis addresses these limitations by integrating data from multiple independent QTL studies to identify consensus QTLs with smaller CI, thereby enhancing the resolution of trait-associated regions. In this study, we report MQTLs for various biotic and abiotic stress resistance traits and key nutritional traits in peanuts, based on 30 QTL studies available in the public domain up to 2024.

## 2 Material and methods

### 2.1 Collection of studies and QTLs

A comprehensive review was conducted to gather information on QTLs associated with abiotic stress, biotic stress, morphological, nutritional, phenological, physiological, and yield-related traits in peanut over the past 12 years. A total of 30 studies were sourced from platforms such as Google Scholar and PubMed. Key details, including population type, logarithm of odds (LOD) score, phenotypic variation explained (PVE/R²), population size, marker positions, QTL locations, and CIs, were extracted from these research articles. Each QTL was treated as an individual entity, even if it was identified in multiple environments or genetic backgrounds. In total, 893 QTLs were initially utilized to conduct the MQTL analysis (**Supplementary Table S1**). All these QTLs were grouped into eight major trait categories (1) aflatoxin (percent seed infection index PSIIA, PSIIB, RAF, AFB2A, AFTA and AFTB), (2) abiotic stress-associated traits (DW, HW, SLA, TR, TE, SDW and ISC), (3) biotic stress-associated traits (AE, ELS, LLS, LS, NH, rust, stem rot, thrips, TSWV, root knot nematodes, NH and bruchid), (4) morphological traits (NB, HRN, IN, NN, PEL, NPB, NSB, IL, PS, TLW, PH, PC, PEL, MSH and PL), (5) nutritional traits (Fe, Zn, OC, OA, AA, BA, GA, LGA, LA, OC, OLR, PA and SA), (6) phenological traits (DE, DF, EDP, TDP, DFF, FI and DW), (7) physiological traits (SCMR, CT, HUE, LDW, NID, SD, LDW, CID, SC, SLW, VCR, RWC and LA), (8) yield related traits (10-SW, HSW, BDW, DPN, HI, PL, DPL, PN, PWE, PWL, PW, PWD, DPW, SN, SWT, SW, SP, SDW, SP and LW). To calculate start CI and end CI positions, we used the following formula for recombinant inbred lines (RIL), back cross (BC) and F_2_ populations (Darvasi and Soller, 1997; Venske et al., 2019; Guo et al., 2006).

For RIL populations, CI = $\frac{163}{P*R2}$ and for F_2_ and BC populations, CI = $\frac{530}{P*R2}$ Where P refers to the size of the population and R^2^ refers to the phenotypic variance explained. The peak, initial, and final positions of the QTLs are also determined for the QTL projection and the MQTL analysis.

### 2.2 Construction of high-density consensus maps

High density consensus maps for all 20 chromosomes were constructed with the help of ‘LPmerge’ package of R software. Markers and their positions from each map of each linkage group were extracted, and ‘.csv’ extension format Excel sheets were prepared as an input file in the R studio. The commands for the LPmerge package were modified according to input data (Endelman and Plomion, 2014). The LPmerge package uses linear programming to efficiently minimize the mean absolute error between the consensus maps and the linkage maps from each population. To obtain the weighted consensus maps, population size of each map was provided in the commands. Then, it creates the “n” number of weighted models, which is selected based on the smallest length of the consensus map and root mean sum of square error (RMSE) value. The best consensus map for all the 20 linkage groups was saved in the ‘.csv’ extension file format.

### 2.3 QTL projection on the consensus maps

The consensus maps constructed using LPmerge was used for MQTL analysis using Biomercator software (Arcade et al., 2004; Olivier et al., 2012; https://mybiosoftware.com/biomercator-genetic-maps-qtl-integration.html). In addition to the consensus map file, QTL information files were also created in ‘.txt’ format. The Veyrieras two-step algorithm (Veyrieras et al., 2007), included in the BioMercator v4.2 software, was used to perform meta-analysis. In the first step, meta-analysis determines the best MQTL model based on model choice criteria from the Akaike information criterion (AIC), a corrected AIC, a Bayesian information criterion (BIC) and the average weight of evidence (AWE). The best MQTL model was selected based on the lowest value and highest weight. All the output information regarding MQTLs and their QTLs from all the 20 linkage groups were extracted. The MQTLs that correspond to weak associations were excluded. MQTL regions containing more than two QTLs for different traits and from different studies were only considered for further analysis. The QTL regions, which deviated from their MQTL positions, were also excluded from the MQTL group. QTL nomenclature is as follows: the name starts with MQTL, followed by the number of the consensus map where the QTL was detected, and a serial number for those MQTLs where two or more were found.

### 2.4 Candidate genes identification from the MQTL region

We used the physical positions of flanking markers from the MQTL regions to retrieve genes from the “NCBI” database for candidate gene identifications. In cases where the physical positions of the flanking markers were not available, the positions of adjacent markers were used instead. Genes were identified using the reference genome assembly of peanut (arahy.Tifrunner.gnm1.KYV3). Genes located in these regions with established functional links to the trait of interest in any crop species were regarded as potential candidate genes for further analysis.

## 3 Results

### 3.1 Salient features of the QTL studies

Thirty independent studies used different types of mapping populations, such as RILs, BC, and F_2_. A total of 893 QTLs were used for the meta-analysis, and all were classified into different categories such as aflatoxin, biotic and abiotic stress-associated traits, morphological, phenological and yield-associated traits (**Supplementary Table S1**). Of these, 30 QTLs related to aflatoxin, 57 QTLs related to abiotic stresses, 158 QTLs related to biotic stresses, 61 QTLs related to morphological traits, 200 QTLs related to nutritional traits, 50 QTLs related to phenological traits, and 87 QTLs related to physiological traits, 250 QTLs related to yield were used in this study. The maximum number of QTLs were observed on LG05 (Linkage Group 05) with 74 QTLs, while the minimum 22 QTLs were on LG17 (**Supplementary Table S2**). The phenotypic variation explained (PVE) varied from 0.013 to 91.1%. Out of 893 QTLs, 437 major QTLs (PVE ≥ 10%) and 456 minor QTLs (PVE ≤10%) were selected for MQTL analysis (**Supplementary Table S1**).

### 3.2 Construction of consensus maps

The consensus map was constructed utilizing pre-existing linkage map data, resulting in a comprehensive map with 11,956 markers spanning 4,496.20 cM (**Supplementary Table S3**). This map contains various types of molecular markers, such as SSRs and SNPs. The distribution of markers per linkage group also showed considerable variation, ranging from 340 markers on LG10 to 1,006 markers on LG05. On an average 2.76 markers per cM were mapped on the consensus genetic map constructed. Linkage group 10 was the shortest, with a length of 128.05 cM, and had the least number of markers. Linkage group 13 was the longest, with a length of 337.9 cM. In addition, we observed substantial variability in marker density across linkage groups (**Supplementary Table S3**). For instance, LG05 has a high marker density of 4.73 markers per cM, while LG02 has a lower density of 1.55 markers per cM, indicating more densely mapped regions in specific chromosomes.

### 3.3 Meta-QTLs detection and their distribution on the peanut genome

**Figure 1:**
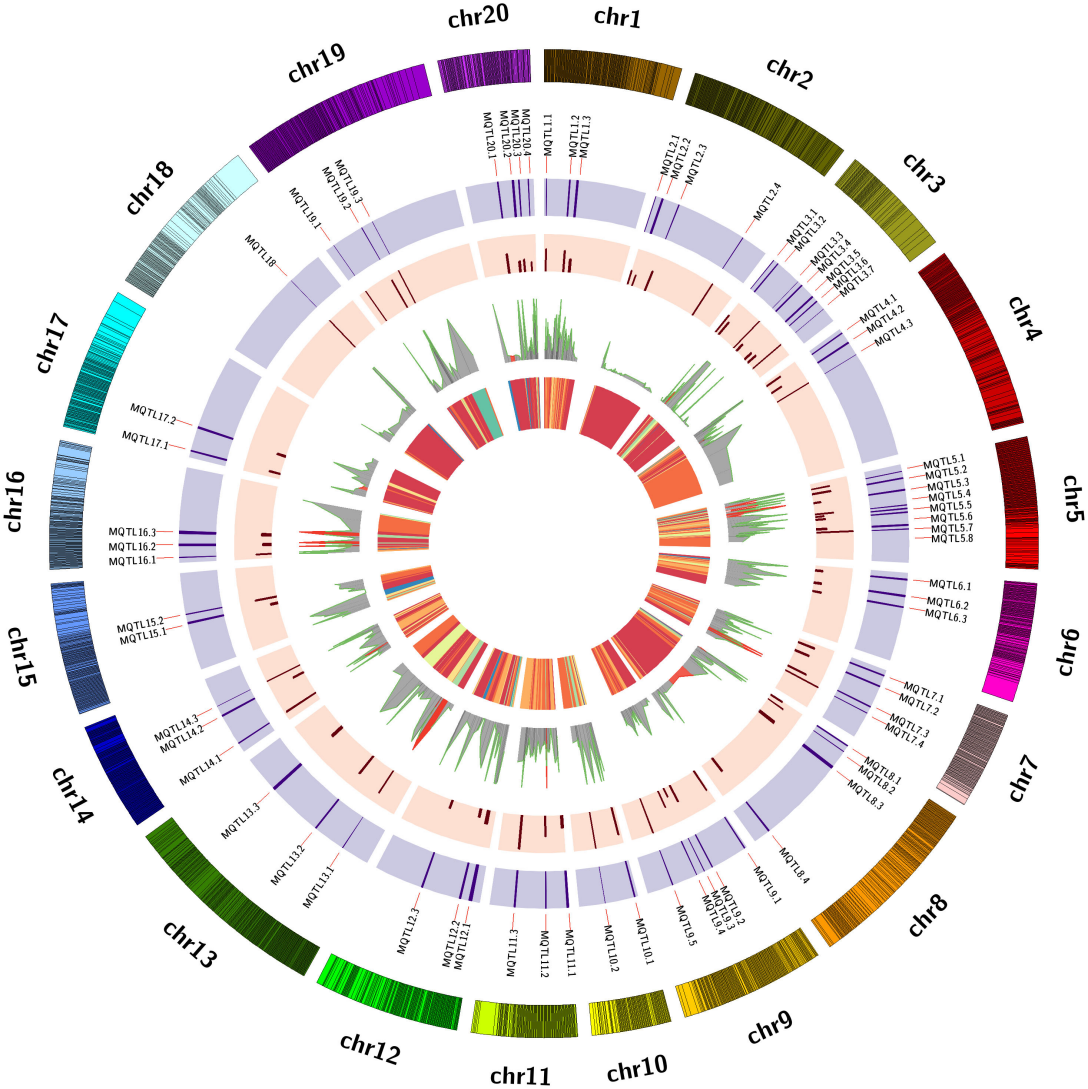
The 70 MQTLs for important traits across 20 chromosomes is illustrated. The circles from inside to outside represent the following: the 1. heatmap of QTLs, 2. projected QTLs with their PVE (Phenotypic Variance Explained), 3. fold change per Meta-QTL, 4. Meta-QTLs with their confidence Intervals (CI), and 5. chromosome wise marker density, respectively.

In the present study, a total of 70 MQTLs were identified across 20 different LGs (**Table 1**). The distribution of MQTLs showed noticeable variability across these regions. LG05 harbored the highest number of MQTLs, with 8 (11.43% of the total), followed by LG03 with 7 MQTLs (10%), and LG09 with 5 MQTLs (7.14%; **Figure 1**). These regions are likely critical for important traits such as disease resistance and seed weight. Other linkage groups, such as LG02, LG07, LG08 and LG20, each contained 4 MQTLs (5.71%), while LG18 had the only single MQTLs (**Supplementary Table S2**). The uneven distribution of MQTLs across the genome points to specific genomic hotspots that may be associated with key traits. Moreover, certain MQTLs, such as MQTL5.4, were associated with many underlying QTLs (15 in this case), which are linked to several traits like late leaf spot (LLS), oil content, and seed size. This suggests that some MQTLs are hotspots for the genetic control of multiple traits, providing an invaluable resource for breeders aiming to improve multiple traits simultaneously. Among the 70 MQTLs, 13 MQTLs contained 18 aflatoxin-associated QTLs. Five aflatoxin QTLs were located on LG05 across three different MQTLs: MQTL5.1, MQTL5.2, and MQTL5.3. Additionally, two QTLs each were identified on LG07 (MQTL7.3), LG13 (MQTL13.1), and LG16 (16.2). MQTL5.2 contains six QTLs associated with palmitic acid, oleic acid, seed weight, seed length and two related to aflatoxin. Similarly, MQTL5.3 grouped two aflatoxin QTLs along with other quality and disease-associated QTLs. MQTL14.1 was crucial for both disease resistance and yield traits, underscoring its role in improving both plant health and productivity. MQTL9.7, MQTL19.1 and MQTL18.8 were significant for enhancing oil content and quality, focusing on the balance of linoleic and oleic acids. MQTL1.4 impacts various morphological traits, offering opportunities to optimize plant structure and pod characteristics. MQTL7.5 addresses physiological traits influencing factors like chlorophyll content and transpiration efficiency, which are vital for plant growth and stress resilience. MQTL10.1 harbors QTLs for multiple traits such as oil content, thrips resistance, leaf spot and rust resistance. Collectively, these MQTLs provide valuable insights for targeted breeding strategies aimed at improving peanut quality, yield, and resilience.

**Table 1:** Meta-QTLs identified on 20 linkage groups of peanut for various traits.

MetaQTL	LG	Position	CI (95%)	CI start	CI end	Left Marker	Right Marker	No. of QTL	Traits
MQTL1.1	1	5.3	1.12	4.74	5.86	5939F	C39	5	TR, Fe, TSWV, SLA and Zn
MQTL1.2	1	56.33	1.78	55.44	57.22	Marker64	bgPabg-595666	8	SLA, PEL, KPA, SDW, PWPP, IN and SP
MQTL1.3	1	73.14	3.34	71.47	74.81	Ah01_38930228	Marker460	5	HUE, LLS, HSW, CT and PW
MQTL2.1	2	0.39	0.5	0.14	0.75	Marker927	Marker893	9	HSW, LW, SL and LW
MQTL2.2	2	15.48	3.58	13.69	5.37	Ah02_98843120	IPAHM524a	4	KDLL, CT and AE
MQTL2.3	2	53.94	1.12	53.38	1.68	GM2196-900	Seq11G07	8	SLA, SP, TW, PYPP, NPPP and PWPP
MQTL2.4	2	207.80	0.28	207.66	0.42	RGC2	Ah5600	8	YOC, PWPP, TDP, HI, TR and HW
MQTL3.1	3	0.35	1.44	0.37	1.07	bgPabg-596210	AX-147243049	8	NPB, PYPP, DFF, SLW, KPA and HSW
MQTL3.2	3	12.03	1.61	11.22	12.83	ARS761-300	PM477	4	SDW, Zn, LS and Fe
MQTL3.3	3	69.72	0.07	69.68	69.75	AX-147217292	AX-147217370	10	MPTO, Fe, WBA, OIL, LLS, NSB and ELS
MQTL3.4	3	81.3	3.53	79.53	83.06	GM2206	PM238-1	4	HUE, PW and HSW
MQTL3.5	3	115.46	2.22	114.35	116.57	AHGS1674	GM2691	3	SLA and SDW
MQTL3.6	3	129.03	2.02	128.02	130.04	AX-147217953	Ah03_133970796	5	NH, VCR and TW
MQTL3.7	3	151.24	0.32	151.08	151.4	Ah03_126798348	Seq15F12	4	SCMR, PW and SW
MQTL4.1	4	10.08	1.21	9.475	10.68	bgPt-593893	bgPt-600898	4	NPPP, TSW and VCR
MQTL4.2	4	34.33	2.61	33.02	35.63	bgPabg-597624	GM890	4	WPA, LLS and TSW
MQTL4.3	4	64.86	0.20	64.76	64.96	AX-147219426	Ah15_155617956	7	Fe, AE, TDP, ISC, PLB and HSW
MQTL5.1	5	15.37	1.20	14.77	15.97	Marker3583	Ah5897	4	LW, SL and KDLL
MQTL5.2	5	30.30	2.20	29.2	31.4	Ah6140	Ah6242	4	WSPA, SL, MPSO and SW
MQTL5.3	5	55.64	2.94	54.17	57.11	Ah4097	Ah5485	3	MPSO and ELS
MQTL5.4	5	88.57	1.44	87.85	89.29	AHGS2534	Ahs2641II	15	YAA, OIL, SP, LLS and PC
MQTL5.5	5	96.46	2.05	95.43	97.485	A05A1146	A05A1355	4	LLS, HPW, AE and PLA
MQTL5.6	5	103.93	1.92	102.97	104.89	AX-147250857	AX-147223064	5	HPW, RLLS, WA and PLA
MQTL5.7	5	131.36	2.86	129.93	132.79	qHW-A05.2	PM112	7	SLA, HW, TE and DW
MQTL5.8	5	140.43	0.19	140.33	140.52	Ah05_114999121	AX-147223487	8	TR, SLA, NB, HW, SDW and DW
MQTL6.1	6	14.31	3.22	12.7	15.92	Ah06_3810427	bgPabg-597436	3	EL, PWA and ELS
MQTL6.2	6	50.12	4.07	48.08	52.15	IPAHM509	Ah06_21266806	3	LLS, NPB and NSB
MQTL6.3	6	74.52	3.85	72.59	76.44	GNB837	TC1A08	4	CT, SP and SCMR
MQTL7.1	7	12.45	2.16	11.37	13.53	bgPabg-594537	seq3B8-400	10	PAA, KPA, SDW, TE, EL, Fe, WSS, SdWA and ISC
MQTL7.2	7	37.54	2.51	36.285	38.795	Marker4573	PM450	11	LDW, GDDFI, HW, CT, SLA, NPB, SDW, PW, SdW and FI
MQTL7.3	7	85.75	1.82	84.84	86.66	Ah6254	AX-147227736	10	WSA, SW, WSPA, NB, ISC, TE and SCMR
MQTL7.4	7	101.29	0.85	100.86	101.71	AHGA102053	GM1986-2	5	PW, DFF, SP and WSB
MQTL8.1	8	2.10	2.38	0.91	3.29	AX-147231702	PM367	4	KDLL, NH and KDrust
MQTL8.2	8	16.51	1.74	15.64	17.38	AX-147229678	Ah6347	5	YPA, DFF, KDrust, RWC and Zn
MQTL8.3	8	58.92	5.78	56.03	61.81	Ahs5872I	AX-147229847	4	YOC, NPPP, KDrust and YLGA
MQTL8.4	8	252.61	2.76	251.23	253.99	TC14B08	A08B47	5	WB, MPTLA, LLS, WPA and MPTO
MQTL9.1	9	3.61	2.06	2.58	4.64	S9_81536603	TC2D08	6	KDrust, TR and KDLL
MQTL9.2	9	84.58	2.12	83.52	85.64	Ah09_117710447	TC1D02	13	PTIFF, PLA, CT, WAA, PW, HUE, TSW and SP
MQTL9.3	9	104.27	1.22	103.66	104.88	Marker5532	AX-147234176	5	OIL, OLE, PAL and LIN
MQTL9.4	9	122.17	1.23	121.55	122.78	AhTE0572	FAD2A	11	MPTL, MPSL, MPTO, YOA, NSB and MPSO
MQTL9.5	9	178.79	0.69	178.44	179.13	Ah6234	Ah6116	5	WSA, SLA, YBA, YLG and YSA
MQTL10.1	10	6.8	2.30	5.66	7.94	TC7H11	Ah6326	6	Thrips, TDP, Kdrust, LS and YOC
MQTL10.2	10	71.98	0.38	71.79	72.16	AhTE0162	Marker6000	7	HW, SP, OLE, SIL, CT and PC
MQTL11.1	11	0.47	9.63	4.34	5.28	Marker6137	seq2G4-1	5	ISC, PLE, LS, TSW and KDLL
MQTL11.2	11	50.05	2.28	48.91	51.19	TOG896615_1198#	SD_c329p435vAC#	14	PW, ELS, DW, TR, SW, PC, HW, LPL and YLGA
MQTL11.3	11	117.84	3.01	116.33	119.34	PM83	PM52	4	SDW, Zn and EDP
MQTL12.1	12	20.54	7.61	16.735	24.34	AHS0046#	AHGS1692_b1	6	DPW, TSW, PLE, DPL and PWD
MQTL12.2	12	42.85	4.26	40.72	44.98	IPAHM531	A02B349	4	SDW, NPPP and MSH
MQTL12.3	12	128.56	2.67	127.22	129.89	Ah12_3356216	GNB1121	4	TW
MQTL13.1	13	61.47	1.11	60.915	62.02	AX-147216965	AX-147252574	9	PTIFF, KDLLS, SW, TE, VCR, HW, SCMR and Zn
MQTL13.2	13	133.4	2.85	131.97	134.82	AX-147217982	Ah2820	9	WSG, MPSO, YOC, Stem Rot, WSP, NPPP, WSL and OIL
MQTL13.3	13	256.94	5.34	254.27	259.61	AHGS1571	RN10F09	3	WG, SN and MSH
MQTL14.1	14	21.11	1.60	20.31	21.91	S14_22478715	GM1959-185	18	TE, SN, MSH, Rust, HW, 10SW, LS, TSW, NPPP, PC, MSH, Fe, Stem Rot and PL
MQTL14.2	14	89.78	3.28	88.14	91.42	AX-147247139	AX-147247229	5	LLS, HSW, MPSO, MPTO and HSW
MQTL14.3	14	112.64	0.08	112.6	112.68	AHGS0202	AX-147219990	9	MPSL, WL, WS, WA, WP, WB, WG, WSS and YPA
MQTL15.1	15	103.97	4.45	101.74	106.19	AX-147250857	Ah15_26507737	3	PW, DFF and FI
MQTL15.2	15	123.2	1.78	122.31	124.09	S1066EaB	AX-147223295	3	FI, PH and DFF
MQTL16.1	16	11.53	1.08	10.99	12.07	PM210	Marker9514	10	PC, HSW, SCMR, PC, SP and SW
MQTL16.2	16	38.66	4.93	36.19	41.12	AX-147226634	S16_74072826	3	ELS, SP and MPTO
MQTL16.3	16	66.21	6.22	63.1	69.32	Ah16_2247722	Ahs3386	3	WP, WA and Stem Rot
MQTL17.1	17	14.89	3.00	13.39	16.39	AX-147227003	AX-147254568	4	DPA, PW, KP and KPA
MQTL17.2	17	67.19	3.50	65.44	68.94	S17_65719159	S17_38129204	3	PL, SP and HSW
MQTL18	18	192.09	0.50	191.84	192.34	GNB159	GM1986-1	9	PTOLR, MPTO, MPTL, WP, MPSO and LLS
MQTL19.1	19	19.11	0.51	18.85	19.36	Marker11860	Ah19_11699918	44	OLA, DFF, PC, LIN, OLE, PL, ShW, CT, PLE and LA
MQTL19.2	19	90.51	0.57	90.22	90.79	FAD2B	AhS67426	10	YPA, MPSL, WSP, MPSLOA, YOC, MPSOLR and YLA
MQTL19.3	19	117.13	0.68	116.79	117.47	gi30420405	AX-147232613	3	YOC and HW
MQTL20.1	20	70.63	2.52	69.37	71.89	Ah20_117411384	AHGS1446	8	Stem Rot, OIL, TSW and TW
MQTL20.2	20	102.84	3.69	100.99	104.68	GM2165	AHGA75537	5	SN, NSB, SP, DW and GDDFI
MQTL20.3	20	116.03	2.03	115.01	117.04	Ah20_143925737	Ah20_126361289	8	HSW, PYPP, GDDFI, SLA, CT and SLA
MQTL20.4	20	136.76	0.69	136.41	137.10	Ah20_143912200	Ah20_134973352	5	HSW, ShW, SP and HI

10SW, Ten seed weight; AA, Arachidic acid; AFB2A, Resistance to production of aflatoxin B2; AE, Adult emergence; AFTA, Aflatoxin content; AFTB, Aflatoxin content; BA, Behenic acid; BC, Backcross; BDW, Biomass dry weight; CI, Confidence interval; CID, Leaf carbon isotope analysis; CT, Canopy temperature; DE, Days of emergence; DF, Days to flowering; DFF, Days to 50% flowering; DPL, Pod length of double seeded; DPW, Pod width of double seeded; DPN, Double seeded pod; DW, Dry weight; EDP, Estimated days to podding; ELS, Early leaf spot; Fe, Iron content; FI, Flower initiation; GA, Gadoleic acid; HI, Harvest index; HRN, Root hairiness; HSW, Hundred seed weight; HUE, Heat use efficiency; HW, Haulm weight; IL, Internode length; ISC, Delta biomass canopy conductance; LA, Leaf area; LDW, Leaf dry weight; LLS, Late leaf spot; LOD, Logarithm of odds; LW, Terminal leaflet width; MQTL, Meta QTL; MSH, Main stem height; NB, Number of branches; NID, Leaf isotope analysis; NH, Number of holes on pod; NN, Node number; NPB, Number of primary branches; NSB, Number of secondary branches; OA, Oleic acid; OC, Oil content; OLR, Oleic to linoleic acid ratio; PA, Palmitic acid; PC, Pod constriction; PEL, Peduncle length; PH, Plant height; PL, Pod length; PN, Pod number per plant; PS, Plant spread; PSIIB, Percent seed infection index for 100 seed weight; PSIIA, Percent seed infection index for 100 seed weight; PSII, Percent seed infection index; PW, Pod weight per plant; PWD, Pod width; PWE, Pod weight; PWL, Pod weight loss; QTLs, Quantitative trait locus; RAF, Aspergillus favus resistance; RILs, Recombinant inbred lines; RWC, Relative water content; SA, Stearic acid; SCMR, SPAD chlorophyll meter reading; SC, Stomatal conductance; SD, Stomatal density; SDW, Shoot dry weight; SDW, Weight of two kernels; SLA, Specific leaf area; SLW, Specific leaf weight; SN, Seed number; SP, Shelling percentage; SWT, Seed weight per plant; TE, Transpiration efficiency; TLW, Terminal leaflet width; TDP, Total developmental period; TR, Transpiration rate; TSWV, Tomato spotted wilt virus; VCR, Visual chlorotic rating; Zn, Zinc content; KPA, Kernel percentage; PWPP, Pod weight per plant; IN, Internode length; KDLL, Late leaf; spot; PYPP, Pod yield per plant; TW, Test weight; NPPP, No of pods per plant; YOC, Oil content; OIL, Oil content; MPTOA, Oleic acid; WBA, Behenic acid; WPA, Palmitic acid; PLB, Pod length; WSPA, Palmitic acid; MPSO, Oleic acid; YAA, Arachidic acid; HPW, Pod weight per plant; RLLS, Late leaf spot; WSS, Stearic acid; GDDFI, Days to flower initiation; WSA, Stearic acid; TE, Transpiration efficiency under WW regime; WSPA, Palmitic acid; WSB, Behenic acid; KDrust, Rust; YLGA, Gadoleic acid; WB, Behenic acid; PAL, Palmitic acid; WSA, Stearic acid; YBA, Behenic acid; YLG, Gadoleic acid; SP, Shelling percentage; SIL, Seed length; PLE, Pod length; LPL, Pod length; YLGA, Gadoleic acid; WSG, Gadoleic acid; WS, Stearic acid; WSS, Stearic acid; PTOLR; Oleic to linoleic acid ratio; MPTO, Oleic acid; MPTL, Linoleic acid; WP, Palmitic acid; MPSO, Oil content; LIN, Linoleic acid content; OLA, Oleic acid; OLE, Oleic acid; MPSLOA, Oleic acid; YOC, Oil content; MPSOLR, Oleic to linoleic acid ratio; ShW, Shell weight.

**Figure 2:**
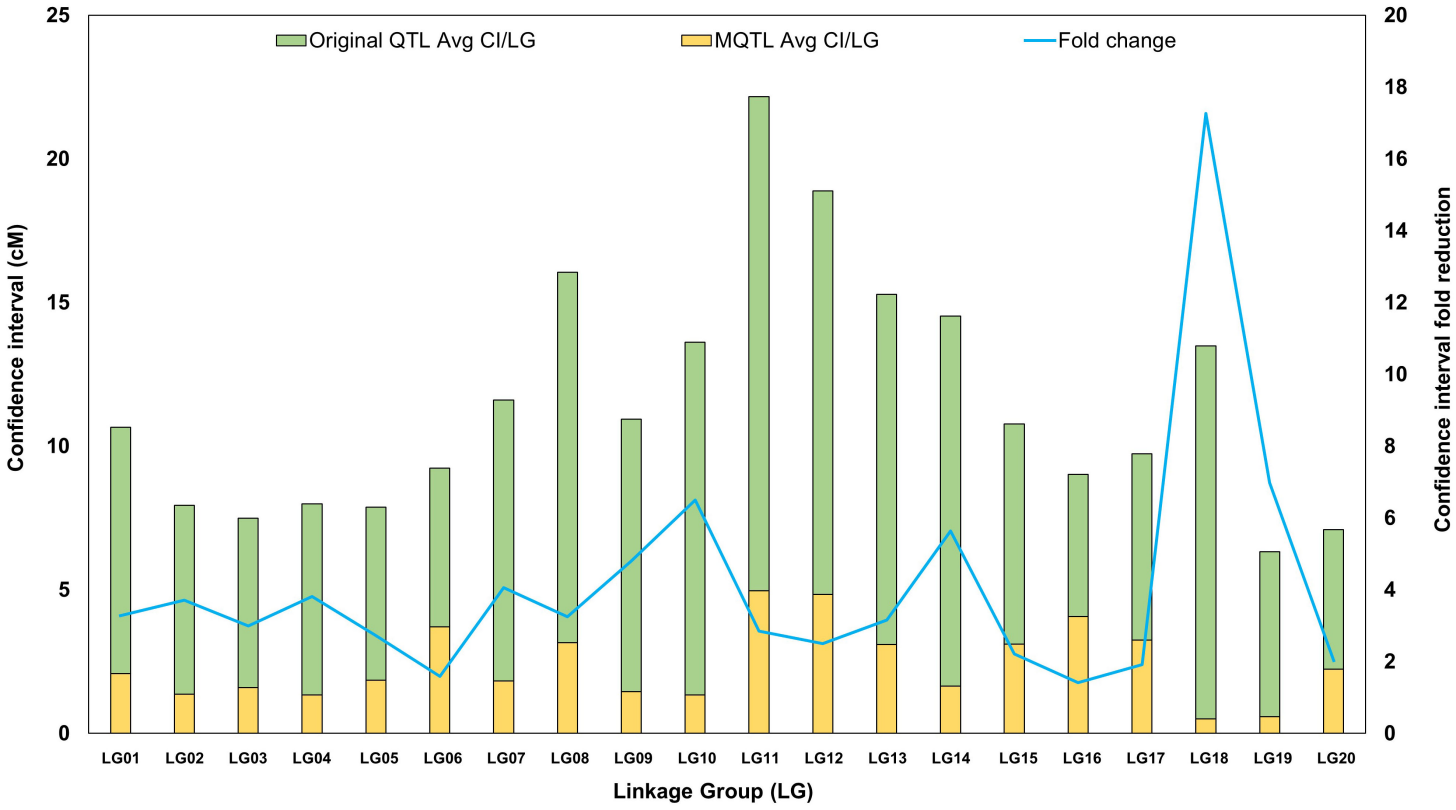
Graphical representation of projected QTLs and Meta-QTLs. Comparison of the mean confidence interval (CI) of original QTLs (green bar) and meta-QTLs (yellow bar). The blue line indicates the average fold reduction in Meta-QTLs.

The precision and stability of the identified MQTLs are evident in their CIs. Initially, the average CIs of the individual QTLs ranged from 1.59 to 29.37 cM, with a mean of 9.26 cM, highlighting variability in the original QTL positions. After meta-analysis, the MQTL CIs significantly decreased, ranging from 0.07 cM (MQTL3.3) to 9.63 cM (MQTL11.1), with an average of 2.33 cM, marking a notable improvement in precision. The fold change in CI, reflects enhanced localization accuracy, varied from 1.76 to 111.38, with an average fold change of 9.02, showcasing substantial improvement in MQTL precision over the original QTLs (**Supplementary Table S4**). Further analysis across linkage groups (LGs) revealed that the average CI of the original QTLs ranged from 5.06 to 17.73 cM, with an overall average of 9.23 cM, indicating considerable variability. After meta-analysis, MQTL CIs were reduced across all LGs, ranging from 0.50 to 4.97 cM, with an average of 2.40 cM (**Supplementary Table S2**; **Figure 2**), demonstrating increased precision across the genome. The fold change in CI reduction also varied across LGs, with LG18 showing the largest fold change (21.58), indicating a substantial improvement in QTL localization. LG10, LG14, and LG19 displayed high fold changes of 8.13, 7.04, and 8.72, respectively, further emphasizing the enhanced precision in these regions (**Supplementary Table S2**). In contrast, LG06 and LG16 had the lowest fold changes, 1.99 and 1.77, respectively, indicating more modest refinements. On average, the fold change across all LGs was 5.17, demonstrating an overall improvement in MQTL localization compared to the original QTLs.

### 3.4 Candidate genes identification

For candidate gene identification, MQTLs less than 50 Mb in size were used. Total 48 MQTL regions were selected for the identification of candidate genes based on the availability of the physical position of the flanking markers of MQTLs (**Supplementary Table S5**). From these meta-genomic regions, we identified a total of 4,024 genes (**Supplementary Table S6**). The functions of these genes were determined through a detailed review of the existing literature. MQTL1.1 is harbors QTLs for transpiration rate, iron content, tomato spotted wilt virus resistance, specific leaf area, and zinc content. For transpiration rate, genes like gibberellin-regulated protein 14-like (*LOC112710127*), caffeoyl-shikimate esterase-like (*LOC112720721*) and EPIDERMAL PATTERNING FACTOR-like protein (*LOC112754391*) were identified. Similarly, gibberellin-regulated protein (*LOC112710127*), transcription factor KUA1 (*LOC112802418*), and several small nuclear RNAs were found to be associated with specific leaf area (Miceli et al., 2019). Disease resistance related genes like serine/threonine-protein kinase (*LOC112801194*), BOI-related E3 ubiquitin-protein ligase (*LOC112801426*), autophagy-related protein (*LOC112802850*), auxin-responsive protein IAA30-like (*LOC112718703*), WAT1-related protein (*LOC112802633*), and protein YABBY 4 (*LOC112803178*) were identified from this region. Iron and zinc content related genes are probable methyltransferases PMT14 (*LOC112801274*), FCS-Like Zinc finger 2 (*LOC112801380*), formyltetrahydrofolate deformylase 1 (*LOC112802537*), ferredoxin C 2 (*LOC112801048*), ion protease homolog 2, peroxisomal (*LOC112801369*), zinc finger A20 and AN1 domain-containing stress-associated protein (*LOC112803256*), transcription factor bHLH18 (*LOC112801016*). MQTL 1.7 is harbors QTLs for the leaf spot, late leaf spot, and rust resistance. We identified some of the important disease resistance related genes from this region like serine/threonine-protein kinase STY13 (*LOC112695569*), ubiquitin-conjugating enzyme E2-23 (*LOC112695673*), WAT1-related protein (*LOC112695708*). On chromosome 3, four MQTL regions were found, and most of the QTLs in this region are yield-related traits. The genes related to these traits are mitogen-activated protein kinase (*LOC112789588*), E3 ubiquitin-protein ligase PUB23 (*LOC112789537*), auxin response factor 6 (*LOC112792550*), DELLA protein GAIP-B (*LOC112792521*). MQTL4.1 contains tomato spotted wilt virus, late leaf spot, and one palmitic acid QTL. F-box/LRR-repeat protein (*LOC112795816*), serine/threonine-protein kinase RUNKEL-like (*LOC112794155*), rust resistance kinase Lr10 (*LOC112795859*), receptor-like protein EIX2 (*LOC112795875*) are mostly related to the disease resistance and acyl carrier protein (*LOC112795780*) is related to the palmitic acid. MQTL5.3 contains disease- and oil-content QTLs. Some important genes related to these traits are proline-rich receptor-like protein kinase (*LOC112802144*), probable polyol transporter (*LOC112802148*), serine acetyltransferase 5-like (*LOC112803828*), serine/threonine-protein kinase phg2 (*LOC112803830*), zinc finger MYM-type protein 1-like (*LOC112803827*), protein FAR-RED IMPAIRED RESPONSE 1-like (*LOC112803829*).

In MQTL8.1, multiple disease-associated QTLs are clustered, and this region contains biotic stress responsive genes such as calcineurin B-like protein (*LOC112708056*), defensin-like protein 1 (*LOC112708031*), and pentatricopeptide repeat-containing protein At3g62890 (*LOC112708064*). On LG09, we generally found MQTLs (MQTL9.7 and MQTL9.3) associated with oil content. However, in MQTL9.7, we also found QTLs for morphological traits in addition to oil content. In MQTL9.3, the genes related to oil traits include fatty-acid desaturase FAD2 (*LOC112710390*), glycerol kinase (*LOC112710344*), linoleate 9S-lipoxygenase (*LOC112710463*), and serine carboxypeptidase (*LOC112710260*). Additionally, in MQTL9.7, genes related to plant growth and development were identified, including VAN3-binding protein (*LOC112711758*), DEAD-box ATP-dependent RNA helicase 7 (*LOC112711726*), ATP sulfurylase 1 (*LOC112711734*), and carboxylesterase 1 (*LOC112711707*). MQTL10.1 contains important genes responsive to biotic stress, including L-type lectin-domain containing receptor kinase VII.1-like (*LOC112715495*), polyadenylate-binding protein RBP47 (*LOC112715552*), serine carboxypeptidase-like 17 (*LOC112715547*), bZIP transcription factor 46 (*LOC112715565*), and zinc finger MYM-type protein 1-like (*LOC112717484*).

MQTL11.2 is the most important region, and it contains 14 QTLs. Squamosa promoter-binding-like protein 6 (*LOC112722612*), serine/threonine-protein phosphatase-7 long form homolog (*LOC112721816*), MADS-box protein (*LOC112722615*) are related to the pod weight. Zinc finger protein (*LOC112721803*), serine protease SPPA, F-box protein (*LOC112722634*) and isoaspartyl peptidase/L-asparaginase (*LOC112722621*) are related to the late leaf spot. Squamosa promoter-binding-like protein (*LOC112722612*), E3 ubiquitin ligase BIG BROTHER-related (*LOC112722633*), serine/threonine-protein phosphatase-7 long form homolog (*LOC112721816*) are related to the dry weight-related traits. Rhamnogalacturonan I rhamnosyl-transferase 1 (*LOC112722626*) and protein breaking of asymmetry in the stomatal lineage-like (*LOC112724059*) are related to the transcription rate and probable UDP-3-O-acylglucosamine N-acyltransferase (*LOC112722632*) is related to gadoleic acid content. These genes are also related to other traits like haulm weight, 100 seed weight and pod constriction. QTLs in MQTL12.1 are related to the pod length and width and disease resistance (Tomato spotted wilt virus). The genes encode for gibberellin 20 oxidase (*LOC112728437*), receptor-like serine/threonine-protein kinase (*LOC112728351*), glyoxylate/hydroxy pyruvate reductase (*LOC112728355*), organelle RRM domain-containing protein (*LOC112728356*), disease resistance protein RML1B (*LOC112728387*), and TMV resistance protein N-like (*LOC112728444*) are mostly related to these traits. MQTL13.1 mostly contains transpiration and chlorophyll related QTLs, protein photosystem I assembly (*LOC112735782*), NDR1/HIN1-like protein (*LOC112737452*), pentatricopeptide repeat-containing protein (*LOC112737396*), protein LOW PSII accumulation 2 (*LOC112737399*), chloroplast envelope quinone oxidoreductase homolog (*LOC112737420*). On LG19, MQTL19.1 harbors QTLs for seed oil content and quality traits. It contains essential genes related to these oil-associated traits, including Acyl-CoA-binding domain-containing protein 6 (*LOC112776181*), fatty Acyl-CoA reductase FAR1 (*LOC112778876*), F-box proteins (*LOC112776494*), and cyclin-dependent kinase inhibitor CDKN1B (*LOC112776306*). MQTL14.3, MQTL18.8, MQTL19.1, MQTL19.4, and MQTL19.5 are found as a hotspot for most of the fatty acid’s QTLs (Lignoceric acid, Stearic acid, Arachidic acid, Palmitic acid, Behenic acid, Gadoleic acid, Stearic acid, Arachidic acid, Oleic acid, Linoleic acid). This region contains multiple copies of oxysterol-binding protein-related protein (*LOC112741888*). Other trait related genes are TBC1 domain family member (*LOC112741897*), glucan endo-1,3-beta-glucosidase (*LOC112741948*), phosphatidylinositol/phosphatidylcholine transfer protein SFH11-like (*LOC112742554*).

## 4 Discussion

Developing high-yielding and climate-resilient crops is crucial for ensuring food and nutritional security. In recent years, various studies have been conducted on peanut to identify genomic regions associated with different important quantitative traits. Despite the identification of thousands of QTLs related to yield and other significant traits in various plant species, only a few have proven useful in genetic improvement programs due to their minor effects and environmental influences. Consequently, the primary objective of this MQTL analysis is to identify stable QTLs in plant genomes that can be effectively utilized in breeding programs through marker-assisted selection.

The distribution and precision of MQTLs across peanut chromosomes offer valuable insights into the genetic architecture of important agronomic traits. Our findings show that certain linkage groups, such as LG01, LG03 and LG05, harbor a higher concentration of MQTLs, indicating these chromosomes as critical regions for traits like disease resistance and seed weight. The presence of up to eight MQTLs on LG05, for instance, highlights its potential as a hotspot for genetic control of these key traits including aflatoxin resistance. The concentration of MQTLs in these linkage groups is comparable to previous studies in other legumes, such as soybean and common bean, where similar clustering of QTLs was observed in specific genomic regions. This suggests that certain chromosomes in legumes may play a more significant role in controlling a variety of traits, thereby making them prime targets for marker-assisted breeding.

A critical aspect of this study is the improved precision of MQTLs, as evidenced by the significant reduction in CIs compared to original QTLs. For example, MQTL3.3, with a CI as narrow as 0.07 cM, indicates a highly stable QTL region, a feature that significantly enhances the accuracy of breeding programs. This improvement in precision is also reflected in the overall reduction of CIs, with many linkage groups showing a fold change greater than four. This mirrors the outcomes of MQTL studies in other legumes, where the refinement of QTL regions has successfully reduced uncertainty around key genomic loci. The narrow CIs in MQTLs provide breeders with more reliable and specific targets, making it easier to incorporate these regions into marker-assisted selection (MAS) programs aimed at improving traits like yield, disease resistance, and seed quality.

In addition to trait-specific MQTLs, the presence of multi-trait MQTLs, such as MQTL5.4 harboring 15 QTLs associated with diverse traits like late leaf spot (LLS), oil content, and seed size, demonstrates the pleiotropic nature of certain genomic regions. This observation is consistent with previous reports in legumes, where specific MQTLs control multiple traits (Kumar et al., 2023). Such regions are particularly valuable in breeding programs as they enable the simultaneous improvement of multiple traits, reducing the need for separate selection processes for each characteristic. Another notable outcome of our study is the variation in marker density across different linkage groups. High-density regions, such as LG10 with 2.83 markers per cM, provide high-resolution genetic maps, which are crucial for fine-mapping QTLs and enhancing the precision of breeding strategies. In contrast, regions with lower marker density, like LG03 with 0.88 markers per cM, may require additional efforts to improve marker saturation for better trait mapping. The observed variability in marker density across linkage groups is consistent with findings in other legume species, where differences in marker distribution often reflect the complexity and evolutionary history of the genome.

Our study also highlights the complexity of nutrition-related traits, which had the highest number of QTLs (200), underscoring the importance of nutritional improvements in crop breeding. This aligns with the growing interest in enhancing nutrient content in legumes, a focus area shared with other legumes like soybean and chickpea, where nutritional traits like protein and oil content have been extensively studied. The identification of numerous QTLs for nutrient traits further emphasizes the potential for genetic improvement in this area, particularly as breeders aim to develop biofortified crops with enhanced nutritional profiles.

In our study, we identified important candidate genes related to the traits in the MQTL region. Total 48 genome-wide MQTLs were selected based on the availability of physical position of the flanking markers. Multiple disease resistance related genes like serine/threonine-protein kinase (*LOC112801194*), *BOI-related E3* ubiquitin-protein ligase (*LOC112801426*), autophagy-related protein (*LOC112802850*), auxin-responsive protein IAA30-like (*LOC112718703*), WAT1-related protein (*LOC112802633*), protein YABBY 4 (*LOC112803178*), zinc finger MYM-type protein 1-like (*LOC112803827*), F-box/LRR-repeat protein (*LOC112795816*), rust resistance kinase Lr10 (*LOC112795859*), and receptor-like protein *EIX2* (*LOC112795875*) were identified from these MQTL regions. Sequencing-based bulk segregant analysis (seq-BSA) combined with nonsynonymous analysis identified the SNP variant in serine/threonine protein kinase gene which has significant role in the fusarium wilt and sterility mosaic disease resistance (Singh et al., 2016). In another study, the mutant population was screened to identify the role of *E3* ubiquitin-protein ligase gene for the disease resistance in rice. The mutant copy of this gene showed a higher expression against the disease infection (You et al., 2016). Transient silencing of the *WAT1* gene in tomato showed resistance to wilt pathogen (Hanika et al., 2021). The over deposition of the F-box proteins was recorded in the nucleus and cytoplasm of the wheat for the immunity activation signaling against the infection leaf rust pathogen (Wei et al., 2020). To protect the bread wheat against biotic stresses like pest and pathogens, wheat breeders have introduced over 200 genes into the cultivated gene pool from various sources. One of the disease resistance genes was tested using the transient expression for its contribution to the resistance against the stem rust. The overexpression of these receptor protein kinases showed resistance against the stem rust of wheat (Yu et al., 2023).

Similarly, transpiration related genes like gibberellin-regulated protein 14-like (*LOC112710127*), caffeoylshikimate esterase-like (*LOC112720721*), EPIDERMAL PATTERNING FACTOR-like protein (*LOC112754391*) and NAC domain-containing protein (LOC112767006) were identified. Gibberellin-regulated protein like DELLA plays a role in signaling the control of the transpiration rate for water use efficiency during the critical phases of plant development and stress management (Locascio et al., 2013). Caffeoylshikimate esterase plays a crucial role in lignin biosynthesis, which provides physical support and water protection by controlling the transpiration network pathway. Due to mutation in this gene and NAC domain, lignin deposition was hampered in one of the droughts related experiments in maize and Arabidopsis (Lu et al., 2013; Sun et al., 2018). Moreover, EPIDERMAL PATTERNING FACTOR-like protein affects the transpiration rate by regulating stomatal density and size. In rice, the overexpression of this gene negatively regulates and reduces the stomatal density, and after this scenario, the experimental lines of rice performed better in terms of their water use efficiency (Caine et al., 2019).

Gibberellin-regulated protein (*LOC112710127*), transcription factor *KUA1* (*LOC112802418*), and many small nuclear RNAs are related to the specific leaf area. GA controls the leaf size by regulating the cell division and increasing water absorption (Ritonga et al., 2023). *KUA* is an *MYB*-like transcription factor that activates leaf expansion and growth-related genes (Schmidt et al., 2016). The overexpression of this gene resulted in a larger cell size, while the mutant of this gene showed a reduction in the size of the cells in leaf tissue in Arabidopsis (Lu et al., 2014).

Iron and zinc content related genes are probable methyltransferase (*LOC112801274*), histone-lysine N-methyltransferase, H3 lysine-9 specific SUVH6 (*LOC112801997*), formyltetrahydrofolate deformylase 1 (*LOC112802537*), ferredoxin C 2 (*LOC112801048*), ion protease homolog 2 (*LOC112801369*), superoxide dismutase (*LOC112719733*) and NAC domain-containing protein (*LOC112767006*). Under iron deficiency, methyltransferase relaxes the chromatin structure for the sufficient expression of *nrf2* gene which regulates iron uptake (Su et al., 2022). Ferredoxin C and superoxidase dismutase is an iron binding protein in plants which also exoculate the transportation of iron (Sharma et al., 2023).

Oil content and fatty acid content related genes such as fatty-acid desaturase *FAD2* (*LOC112710390*), glycerol kinase (*LOC112710344*), linoleate 9S-lipoxygenase (*LOC112710463*), serine carboxypeptidase (*LOC112710260*), oxysterol-binding protein-related protein (*LOC112741888*), TBC1 domain family member (*LOC112741897*), glucan endo-1,3-beta-glucosidase (*LOC112741948*), phosphatidylinositol/phosphatidylcholine transfer protein SFH11-like (*LOC112742554*). Glycerol kinase provides the precursors to the lipid biosynthesis. The overexpression of this gene showed the increase in lipid production and showed the resistance to bacterial blight and blast diseases in rice (Xiao et al., 2022). Fatty-acid desaturase plays a crucial role lipid biosynthesis and for converting mono-unsaturated fatty acids into poly unsaturated fatty acids which is important for the normal development and function of plants (Czumaj and Śledziński, 2020). This enzyme also helps with other proteins in the determining the oil content and composition in the seeds. Linoleate 9S-lipoxygenase uses linoleic acid or linolenic acid as a substrate and gets involved in plant development and growth, stress tolerance, and senescence (Vellosillo et al., 2007). Phosphatidylinositol/phosphatidylcholine transfer protein and oxysterol-binding protein are also a lipid binding protein and play various roles in plant metabolism and stress response (Lete et al., 2020; Ye et al., 2022). The overexpression of the serine carboxypeptidase gene in the Arabidopsis negatively regulates and reduces the production of membrane lipid (Chen, 2020).

A few more genes related to plant growth and development were identified from different MQTL regions, including VAN3-binding protein (*LOC112711758*), DEAD-box ATP-dependent RNA helicase 7 (*LOC112711726*), ATP sulfurylase 1 (*LOC112711734*), and carboxylesterase 1 (*LOC112711707*). VAN3-binding protein activates proteins which regulate auxin transport mediated pathways and leads to continuous venation and root elongation (Naramoto and Kyozuka, 2018). DEAD-box ATP-dependent RNA helicase plays a role in plant growth, development, and found to be upregulated in abiotic stresses of wheat (Ru et al., 2021). ATP sulfurylase is important for Sulphur assimilation in plants. Sulphur is an essential macronutrient for the growth and development of plants (Anjum et al., 2015). Carboxylesterase also plays an important role in the growth, development, and stress tolerance in plants. The cis element of this gene was mostly found to be related to plant hormones like GA and IAA and the expression of this gene was mostly found in the root, leaf and stem of cotton (Rui et al., 2022).

In our study, while identifying candidate genes we have considered and highlighted the genes that are reported to play a key role in peanut as well as other key crops. For instance, genes encoding NAC domain-containing protein in the hotspot region was reported to play key role in biotic, abiotic, nutritional, and physiological traits (Yuan et al., 2020; Li et al., 2021; Yuan et al., 2023). In addition, we identified key genes associated with aflatoxin resistance in MQTL5.2 (serine/threonine-protein kinase, BOI-related E3 ubiquitinprotein ligase), MQTL5.3, MQTL7.3, and MQTL13.1. Comparative proteomic studies indicated that serine/threonine-protein kinase was reported to play a role in aflatoxin resistance in maize (Chen et al., 2005; Brown et al., 2010). While global transcriptome profiling studies reported that BOI-related E3 ubiquitinprotein ligase plays a role on pre-harvest aflatoxin contamination in peanut. Similarly, for yield-related traits in MQTL3.1-MQTL3.4 (mitogen-activated protein kinase, auxin response factor), MQTL11.2 (MADS-box protein, squamosa promoter-binding protein), and MQTL14.1. *AhMPK3* exists in two copies in peanut genome and its structural organization revealed well-conserved nature of these signaling components across different species (Kumar et al., 2009). Further, auxin response factor *AhARF6* reported to play a role in peanut pod development (Li et al., 2021). In case of peanut, thirty-eight genes (*AhSPL1*-*AhSPL38*) were identified and *AhSPL* genes might be widely involved in peanut growth and development, as well as in response to environmental stresses (Sun et al., 2024). Several genes like fatty-acid desaturase FAD2, linoleate 9S-lipoxygenase, acyl-CoA-binding protein are reported to play role in the oil composition and some of these genes were edited and cloned (Peng et al., 2020; Neelakandan et al., 2022; Zhao et al., 2022). Genes related to oil composition within MQTL5.2 (fatty-acid desaturase FAD2, linoleate 9S-lipoxygenase), MQTL9.3, MQTL19.1 (acyl-CoA-binding protein, fatty acyl-CoA reductase FAR1), MQTL19.4, and MQTL19.5. Nutritional traits like iron and zinc content are linked to MQTL1.1 (probable methyltransferase, ferredoxin C), MQTL10.1, and MQTL12.1.

## 5 Conclusion

In conclusion, the outcomes of this study demonstrate the power of MQTL analysis in refining genomic regions associated with important traits. The reduction in QTL CIs, the identification of multi-trait MQTLs, and the concentration of MQTLs on key linkage groups provide valuable insights that can accelerate breeding efforts in peanut. These findings are consistent with MQTL studies in other legumes, suggesting a broader applicability of this approach across legume species. Future research should focus on further refining MQTL regions and increasing marker density in less-dense genomic regions to ensure comprehensive coverage of trait-associated loci, ultimately improving the effectiveness of MAS in crop breeding programs. Furthermore, we found that nutritional-related traits had the highest number of initial QTLs (200), reflecting their genetic complexity and the substantial research aimed at enhancing nutrient content in crops. Other categories, such as biotic traits (156 QTLs) and abiotic traits (57 QTLs), were also prominent, showing the broad scope of focus in breeding programs. When compared with previously reported MQTL studies in legumes, similar trends of concentration of MQTLs on key chromosomes and the improvement in QTL precision were observed. For example, studies on soybean and common bean also highlighted that specific chromosomes harbor denser MQTLs, particularly for traits like disease resistance and yield. The reduction in CIs in peanut mirrors findings in other legumes, indicating that MQTL analysis consistently enhances precision across legume species, providing breeders with robust, reliable genomic regions for trait improvement.

## Data Availability

The original contributions presented in the study are included in the article/**Supplementary Material**. Further inquiries can be directed to the corresponding author/s.
