# Supplementary material for: Consensus genomic regions and key genes for biotic, abiotic and key nutritional traits identified using meta- QTL analysis in peanut
Source: Front Plant Sci. 2025 Apr 15;16:1539641. doi: 10.3389/fpls.2025.1539641 (PMC12038908; doi:10.3389/fpls.2025.1539641)
Supplement: Supplementary file 1 [file DataSheet.docx]

Ahmad, S., Nawade, B., Sangh, C., Mishra, G. P., Bosamia, T. C., Kumar, N., et al. (2020). Identification of novel QTLs for late leaf spot resistance and validation of a major rust QTL in peanut (*Arachis hypogaea* L.). *Biotech* 10, 458. doi:10.1007/s13205 020-02446-4

Burow, M. D., Starr, J. L., Park, C. H., Simpson, C. E., and Paterson, A. H. (2014). Introgression of homeologous quantitative trait loci (QTLs) for resistance to the root-knot nematode (*Meloidogyne arenaria*) in an advanced backcross-QTL population of peanut (*Arachis hypogaea* L.). *Mol. Breed.* 34, 393–406. doi:10.1007/s11032-014-0042-2

Chai, H. H., Ho, W. K., Graham, N., May, S., Massawe, F., and Mayes, S. (2017). A cross-species gene expression marker-based genetic map and QTL analysis in Bambara groundnut. *Genes* 8, 84. doi:10.3390/genes8020084

Chavarro, C., Chu, Y., Corley Holbrook, T., Isleib, T., Bertioli, D., Hovav, R., et al. (2020). Pod and seed trait QTL identification to assist breeding for peanut market preferences. *G3 Genes|Genomes|Genetics* 10, 2297–2315. doi:10.1534/g3.120.401147

Dodia, S. M., Joshi, B., Gangurde, S. S., Thirumalaisamy, P. P., Mishra, G. P., Narandrakumar, D., et al. (2019). Genotyping-by-sequencing based genetic mapping reveals large number of epistatic interactions for stem rot resistance in groundnut. *Theor. Appl. Genet.* 132, 1001–1016. doi:10.1007/s00122-018-3255-7

Faye, I., Pandey, M. K., Hamidou, F., Rathore, A., Ndoye, O., Vadez, V., et al. (2015). Identification of quantitative trait loci for yield and yield-related traits in groundnut (*Arachis hypogaea* L.) under different water regimes in Niger and Senegal. *Euphytica* 206, 631–647. doi:10.1007/s10681-015-1472-6

Ghosh, S., Mahadevaiah, S. S., Gowda, S. A., Gangurde, S. S., Jadhav, M. P., Hake, A. A., et al. (2022). Genetic mapping of drought tolerance traits phenotyped under varying drought stress environments in peanut (*Arachis hypogaea* L.). *Euphytica* 218, 168. doi:10.1007/s10681-022-03120-x

Hake, A. A., Shirasawa, K., Yadawad, A., Sukruth, M., Patil, M., Nayak, S. N., et al. (2017). Mapping of important taxonomic and productivity traits using genic and non-genic transposable element markers in peanut (*Arachis hypogaea* L.). *PloS One* 12, e0186113. doi:10.1371/journal.pone.0186113

Jadhav, M. P., Gangurde, S. S., Hake, A. A., Yadawad, A., Mahadevaiah, S. S., Pattanashetti, S. K., et al. (2021). Genotyping-by-sequencing based genetic mapping identified major and consistent genomic regions for productivity and quality traits in peanut. *Front. Plant Sci*. 12. doi:10.3389/fpls.2021.668020

Jiang, Y., Luo, H., Yu, B., Ding, Y., Kang, Y., Huang, L., et al. (2021). High-density genetic linkage map construction using whole-genome resequencing for mapping QTLs of resistance to *Aspergillus flavus* infection in peanut. *Front. Plant Sci*. 12. doi:10.3389/fpls.2021.745408

Jin, G., Liu, N., Yu, B., Jiang, Y., Luo, H., Huang, L., et al. (2023). Identification and pyramiding major QTL loci for simultaneously enhancing aflatoxin resistance and yield components in peanut. *Genes* 14, 625. doi:10.3390/genes14030625

Khan, S. A., Chen, H., Deng, Y., Chen, Y., Zhang, C., Cai, T., et al. (2020). High density SNP map facilitates fine mapping of QTLs and candidate gene discovery for *Aspergillus flavus* resistance in peanut (*Arachis hypogaea*). *Theor. Appl. Genet.* 133, 2239–2257. doi:10.1007/s00122-020-03594-0

Khedikar, Y. P., Pandey, M. K., Sujay, V., Monyo, E., Hoisington, D. A., Guo, B., et al. (2018). Identification of main effect and epistatic quantitative trait loci for morphological and yield-related traits in peanut (*Arachis hypogaea* L.). *Mol. Breed.* 38, 7. doi:10.1007/s11032-017-0764-z

Leal-Bertioli, S. C. M., Moretzsohn, M. C., Roberts, P. A., et al. (2016). Genetic mapping of resistance to *Meloidogyne arenaria* in *Arachis stenosperma*: A new source of nematode resistance for peanut. *G3 Genes|Genomes|Genetics* 6, 377–390. doi:10.1534/ g3.115.023044

Luo, H., Guo, J., Ren, X., Chen, W., Huang, L., Zhou, X., et al. (2018). Chromosomes A07 and A05 associated with stable and major QTLs for pod weight and size in cultivated peanut (*Arachis hypogaea* L.). *Theor. Appl. Genet.* 131, 267–282. doi:10.1007/s00122-017-3000-7

Mondal, S., Hadapad, A. B., Hande, P. A., and Badigannavar, A. M. (2014). Identification of quantitative trait loci for bruchid (*Caryedon serratus* Olivier) resistance components in cultivated groundnut (*Arachis hypogaea* L.). *Mol. Breed.* 33, 961–973. doi:10.1007/s11032-013-0011-1

Pandey, M. K., Wang, M. L., Qiao, L., and Qin, H. (2014). Identification of QTLs associated with oil content and mapping FAD2 genes and their relative contribution to oil quality in peanut (*Arachis hypogaea* L.). *BMC Genet*. 15, 133. doi:10.1186/s12863 014-0133-4

Pandey, M. K., Wang, H., Khera, P., Vishwakarma, M. K., Kale, S. M., Culbreath, A. K., et al. (2017). Genetic dissection of novel QTLs for resistance to leaf spots and tomato spotted wilt virus in peanut (*Arachis hypogaea* L.). *Front. Plant Sci.* 8. doi: 10.3389/fpls.2017.00025

Pandey, M. K., Gangurde, S. S., Sharma, V., Pattanashetti, S. K., Naidu, G. K., Faye, I., et al. (2021). Improved genetic map identified major QTLs for drought tolerance and iron deficiency tolerance-related traits in groundnut. *Genes* 12, 37. doi: 10.3390/genes12010037

Parmar, S., Janila, P., Gangurde, S. S., Variath, M. T., Sharma, V., Bomireddy, D., et al. (2023). Genetic mapping identified major main-effect and co-localized QTLs controlling high iron and zinc content in groundnut. *Plant Genome* 16, e20361. doi:10.1002/tpg2.20361

Pattanashetti, S. K., Pandey, M. K., Naidu, G. K., Vishwakarma, M. K., Singh, O. K., Shasidhar, Y., et al. (2020). Identification of quantitative trait loci associated with iron deficiency chlorosis resistance in groundnut (*Arachis hypogaea*). *Plant Breed.* 139, 790-803. doi:10.1111/pbr.12833

Qin, H., Feng, S., Chen, C., Guo, Y., Knapp, S., Culbreath, A., et al. (2012). An integrated genetic linkage map of cultivated peanut (*Arachis hypogaea* L.) constructed from two RIL populations. *Theor. Appl. Genet.* 124, 653–664. doi:10.1007/s00122-011 1737-y

Sharma, V., Gangurde, S. S., Nayak, S. N., et al. (2023). Genetic mapping identified three hotspot genomic regions and candidate genes controlling heat tolerance-related traits in groundnut. *Front. Plant Sci.* 14. doi:10.3389/fpls.2023.1182867

Shasidhar, Y., Vishwakarma, M. K., Pandey, M. K., et al. (2017). Molecular mapping of oil content and fatty acids using dense genetic maps in groundnut (*Arachis hypogaea* L.). *Front. Plant Sci.* 8. doi:10.3389/fpls.2017.00794

Sujay, V., Gowda, M. V. C., Pandey, M. K., et al. (2012). Quantitative trait locus analysis and construction of consensus genetic map for foliar disease resistance based on two recombinant inbred line populations in cultivated groundnut (*Arachis hypogaea* L.). *Mol. Breed.* 30, 773–788. doi:10.1007/s11032-011-9661-z

Tayade, A. D., Motagi, B. N., Jadhav, M. P., et al. (2022). Genetic mapping of tolerance to iron deficiency chlorosis in peanut (*Arachis hypogaea* L.). *Euphytica* 218, 46. doi:10.1007/s10681-022-02973-0

Wang, H., Wang, H., Pandey, M. K., et al. (2013). Genetic mapping and quantitative trait loci analysis for disease resistance using F2 and F5 generation-based genetic maps derived from ‘Tifrunner’ × ‘GT-C20’ in peanut. *Theor. Appl. Genet.* 126, 1173–1190. doi:10.1007/s00122-013-2043-0

Wang, M. L., Khera, P., Pandey, M. K., et al. (2015). Genetic mapping of QTLs controlling fatty acids provided insights into the genetic control of fatty acid synthesis pathway in peanut (*Arachis hypogaea* L.). *PloS One* 10, e0119454. doi: 10.1371/ journal.pone.0119454

Yu, B., Huai, D., Huang, L., Kang, Y., Ren, X., Chen, Y., et al. (2019). Identification of genomic regions and diagnostic markers for resistance to aflatoxin contamination in peanut (*Arachis hypogaea* L.). *BMC Genet.* 20, 32. doi:10.1186/s12863-019-0734-z

Zhang, S., Hu, X., Miao, H., Chu, Ye., Cui, F., Yang, W., et al. (2019). QTL identification for seed weight and size based on a high-density SLAF-seq genetic map in peanut (*Arachis hypogaea* L.). *BMC Plant Biol.* 19, 537. doi.org/10.1186/s12870-019-2164-5
